# Fostering Conservation via an Integrated Use of Conventional Approaches and High-Throughput SPET Genotyping: A Case Study Using the Endangered Canarian Endemics *Solanum lidii* and *S. vespertilio* (Solanaceae)

**DOI:** 10.3389/fpls.2020.00757

**Published:** 2020-07-10

**Authors:** Pietro Gramazio, Ruth Jaén-Molina, Santiago Vilanova, Jaime Prohens, Águedo Marrero, Juli Caujapé-Castells, Gregory J. Anderson

**Affiliations:** ^1^Faculty of Life and Environmental Sciences, University of Tsukuba, Tsukuba, Japan; ^2^Instituto de Conservación y Mejora de la Agrodiversidad Valenciana, Universitat Politècnica de València, Valencia, Spain; ^3^Jardín Botánico Canario “Viera y Clavijo” – Unidad Asociada al CSIC, Cabildo de Gran Canaria, Las Palmas de Gran Canaria, Spain; ^4^Department of Ecology and Evolutionary Biology, University of Connecticut, Storrs, CT, United States

**Keywords:** conservation, endangered endemics, reproductive biology, SNPs, Solanaceae, *Solanum*, Canary Islands, SPET

## Abstract

Islands provide unique opportunities to integrated research approaches to study evolution and conservation because boundaries are circumscribed, geological ages are often precise, and many taxa are greatly imperiled. We combined morphological and hybridization studies with high-throughput genotyping platforms to streamline relationships in the endangered monophyletic and highly diverse lineage of *Solanum* in the Canarian archipelago, where three endemic taxa are currently recognized. Inter-taxa hybridizations were performed, and morphological expression was assessed with a common-garden approach. Using the eggplant Single Primer Enrichment Technology (SPET) platform with 5,093 probes, 74 individuals of three endemic taxa (*Solanum lidii*, *S. vespertilio* subsp. *vespertilio*, and *S. vespertilio* subsp. *doramae*) were sampled for SNPs. While morphological and breeding studies showed clear distinctions and some continuous variation, inter-taxon hybrids were fertile and heterotic for vigor traits. SPET genotyping revealed 1,421 high-quality SNPs and supported four, not three, distinct taxonomic entities associated with post-emergence geological, ecological and geographic factors of the islands. Given the lack of barriers to hybridization among all the taxa and their molecular differences, great care must be taken in population management. Conservation strategies must take account of the sexual and breeding systems and genotypic distribution among populations to successfully conserve and restore threatened/endangered island taxa, as exemplified by *Solanum* on the Canary Islands.

## Introduction

Islands have long been recognized as biological laboratories because they provide particularly insightful understanding into the evolution, ecology, and conservation of plant species (Baldwin and Warren, 2010; Caujapé-Castells et al., 2010; Warren et al., 2015; Weigelt et al., 2015; Nogales et al., 2016; Stuessy et al., 2017a; Price et al., 2018). Their geologic ages can also be determined fairly precisely, thereby making it possible to infer approximate colonization times associated with diversification events, including, sometimes, the order of colonization (Curto et al., 2017). When coupled with phylogenies thoroughly sampled geographically and genetically, such methodological advances have led to an improved understanding of tempos and patterns of speciation (Mairal et al., 2015). Furthermore, when the biology of the taxa is well studied, including reproductive systems, the inference of drivers of evolution is possible, and conservation and restoration programs can be developed for populations that are often vulnerable and small (Crawford et al., 2019).

Notably, a number of molecular-based studies have revealed sharp genetic discontinuities within and between islands in both narrowly distributed and widespread Canarian endemic plant lineages (Rumeu et al., 2014; Jaén-Molina et al., 2015; Puppo et al., 2015; García-Verdugo et al., 2017). These results emphasize the value of comprehensive sampling of populations and genomes for a thorough understanding of evolution prior to the implementation of the necessary components of an effective conservation strategy (Caujapé-Castells et al., unpublished; Díaz-Bertrana and Saturno, 2017).

*Solanum lidii* Sunding and *S. vespertilio* Aiton are endangered endemic Solanaceae from the Canary Islands that are notable for their suite of reproductive features [(e.g., andromonoecious, self-compatible, heterandrous, sometimes four-parted, always zygomorphic, and sometimes enantiostylous (Anderson et al., 2015)]. Furthermore, molecular genetic studies of these species (Prohens et al., 2007; Caujapé-Castells et al., unpublished) revealed unexpected patterns of variation that have promoted a significant investment in their conservation, particularly on Gran Canaria (Mesa-Coello et al., 2013).

**FIGURE 1:**
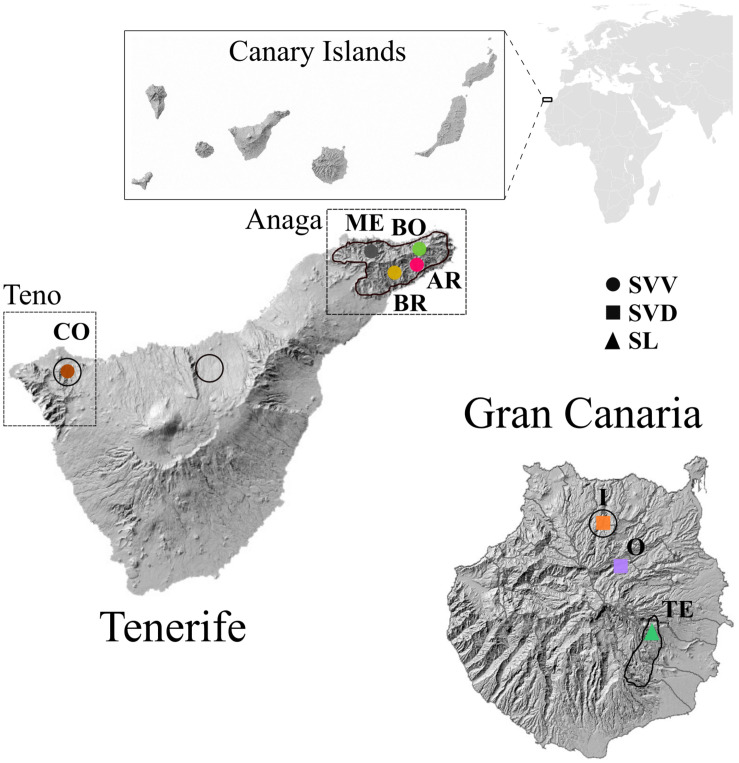
Wild distribution of the three Canarian endemic *Solanum* taxa in Tenerife and Gran Canaria highlighted in the map with a black line, following Rodríguez and Garzón (2019) and https://www.biodiversidadcanarias.es/. Populations from the same taxa are represented with the same shape but different colors, following codes as in Table 1.

*Solanum lidii* (hereafter referred to as SL) is endemic to Gran Canaria, where it grows in ruderal shrublands on disturbed sites and rocky areas as inland cliffs (Rodríguez-Delgado et al., 2004) at between 350 and 725 m. In 2004, the total size of SL in the wild was estimated at only 93 individuals, distributed among six isolated and severely fragmented subpopulations in five locations. The area around Temisas, in the southeast of Gran Canaria (Figure 1) is the largest but includes only 32 individuals (Rodríguez-Delgado et al., 2004). Intrusive human development, changes in land use, collapse of terrain (or landslides) and herbivory (often by goats) are the main threats to the continued existence of this species (Rodríguez-Delgado et al., 2004). SL is listed under the category “Critically Endangered” by the IUCN criterion [CR B2ab (ii, iii)]; C2a(i) in the Spanish Red List 2008 (Moreno, 2008), and as a species in danger of extinction on the Spanish national and regional catalogs of threatened species^1^. Among the proposed measures to reduce extinction risks for SL are the construction of fences and the local elimination of goat grazing.

**TABLE 1:** Samples and population details of the *Solanum* taxa studied: *S. vespertilio* subsp. *vespertilio* (SVV), *S. vespertilio* subsp. *doramae* (SVD), and *S. lidii* (SL).

Population	Accessions	Taxon	Locality	Island	LPA herbarium code	DNA bank code
SVV_BR	1–9	SVV	Valle Brosque, Anaga	T	36864–36865	20532–20544
SVV_BO	1–18	SVV	Lomo de Las Bodegas, Anaga	T	36868	20558–20574
SVV_AR	1–7	SVV	Cabezo de Arbei, Anaga	T	36866–36867	20550–20557
SVV_ME	1–10	SVV	Mesa del Brezal, Anaga	T	36871	20575–20580
SVV_CO	1–15	SVV	Barranco de Los Cochinos, Teno	T	36872–36873	20581–20601
SVD_I	1	SVD	IES Doramas, Moya (ex horto El Arco*)	C	37538–37539	20461
SVD_O	1–4	SVD	Finca de Osorio, Teror (ex horto*)	C	37580–37581	20456–20459
SL_TE	1–10	SL	Lomo de La Cruz, Temisas	C	37521–37522	20464–20505

*Canary Islands: T, Tenerife; C, Gran Canaria. ^∗^From seeds of the natural population of Barranco de Azuaje.*

*Solanum vespertilio* includes two subspecies, *S. vespertilio* subsp. *vespertilio* (hereafter SVV) and *S. vespertilio* subsp. *doramae* (hereafter SVD) Marrero Rodr. & Gonzalez-Martin. SVV is restricted to Tenerife, the largest of the seven main islands in the Canarian archipelago, where the greatest concentration of individuals occurs in two areas: the palaeo-islands of Teno (in the northwest; a dryer habitat) and Anaga (in the northeast; a moister habitat) (Figure 1). While only one population is found in Teno, the Anaga area includes at least five populations scattered throughout several ravines. The main threats to this subspecies are competing exotic species, goat herbivory and parasitism. It is included as “Vulnerable to changes in its habitat” in the Canarian Catalogue of Endangered Species (2001) and considered as “Critically endangered” by the IUCN [CR B2ab (iii, v)] in the Spanish Red List 2008 (Moreno, 2008). Proposed conservation measures include local elimination of goat grazing and supplemental planting within the current distribution, and in other similar habitats.

SVD is a very rare, and possibly extinct in the wild, endemic of Gran Canaria. It was cited first from just two localities by Webb and Berthel (1845); Kunkel (1975a) considered it extinct. However, a population of around 15 individuals was rediscovered in “Barranco de Azuaje” (Marrero, 1986). Based on morphological and ecological differences, the Gran Canaria individuals were recognized as a new subspecies: *S. vespertilio* subsp. *doramae* (Marrero and González Martín, 1998). Since 1987, four censuses (Marrero and González Martín, 1998 and SEGAs 2002, 2005, and 2006) have reported the decline of SVD, first to six and later to only 2–3 mature plants (Santana and Naranjo, 2002; Docoito-Díaz and Herrera, 2005; Cabrera et al., 2006). The decline to near-extinction of SVD was mainly due to agriculture, including grazing, and to the competition with introduced exotics (e.g., *Agave americana* and various Opuntia, Bañares et al., 2003). In 2000, SVD was included under the category “endangered” in the National Catalogue of Threatened Species and the following year, in the Canarian Catalogue of Endangered Species. In 2008, the Canarian Government approved a recovery plan for this subspecies.

In the most recent molecular study of the relationships among these three taxa, Caujapé-Castells et al. (unpublished data) assessed five cpDNA sequences (*matK, rbcL*, *trnT-L*, *trnK-rps16*, and *trnF-L*) and ISSR markers. That study, conducted on limited population sampling (five individuals of each of SVV and SVD and three SL), aimed to quantify the genetic differentiation among the three taxa to assess the potential for reintroduction of the threatened SVD in its natural habitats on Gran Canaria. Despite the relatively high levels of polymorphism detected with both molecular techniques, the barcode sequences (*matK* and *rbcL*) did not reveal distinction between the two subspecies of *S. vespertilio*, and only one position in *trnL-F* discriminated *S. lidii* from *S. vespertilio*. These results reinforced the close phylogenetic relationship among the taxa highlighted in the AFLP analysis by Prohens et al. (2007) but also indicated the need for future multi-disciplinary studies that included molecular approaches with greater discriminatory power.

Single nucleotide polymorphisms (SNPs) are among the most abundant and easy to automate molecular markers (Thomson, 2014; Kim et al., 2016). As a result of the tremendous advances in next-generation sequencing (NGS) platforms and the decrease in sequencing costs, it has become easier to identify hundreds of thousands or even million SNPs in a short time and at a reasonable cost (Scheben et al., 2017). Recently, the first SNP high-throughput genotyping platform (specifically designed for eggplant and its wild relatives, Barchi et al., 2019a) was developed, derived from the resequencing of seven *S. melongena* and one *S. incanum* accessions (Gramazio et al., 2019). The eggplant SPET platform is based on 5,093 target SNPs distributed throughout the whole eggplant genome. Barchi et al. (2019a) pointed out the suitability and transferability of the eggplant SPET platform to closely related species as a result of the discovery of novel SNPs in the surrounding sequenced regions of the target probes. Particularly relevant for this study, the threatened taxa at issue are closely related to the eggplant group (Prohens et al., 2007).

Traditional and new molecular tools have been revealed as powerful and indispensable aids to conservation efforts, particularly with endangered species (Corlett, 2017). Herein, we combine data from traditional studies of morphological expression in common garden experiments with experimental plant hybridizations, and the latest DNA technology (via high-throughput SNP genotyping using the eggplant SPET platform), to substantiate conservation strategies for rare and endangered island plants. We believe our multi-faceted approach will help clarify the possible genetic origin and stability of the morphological characters separating these taxa at a level not previously possible (Prohens et al., 2007; Särkinen et al., 2013). The main aims of this study were to (i) study the morphological differences of the Canarian endemics *S. lidii* and *S. vespertilio s.l.*, and their crossability, (ii) provide new data to assess their genetic relationships and (iii) suggest effective conservation strategies for these endangered taxa, that include the extinct-in-the wild *S. vespertilio* ssp. *doramae*.

## Materials and Methods

### Plant Material

A total of 74 individuals from the three *Solanum* taxa were sampled (Table 1). Leaves were collected during 2018 and dried and conserved in silica gel for subsequent analysis. The bags with dried leaves were deposited in the DNA Bank of the Canarian Flora at Jardín Botánico Canario “Viera y Clavijo” – Unidad Asociada al CSIC of the Cabildo de Gran Canaria (JBCVCSIC hereafter) and vouchers for each taxon and population were collected and deposited at the LPA herbarium at JBCVCSIC. Samples were collected in eight localities that encompass their main wild areas of distribution in Gran Canaria and Tenerife, save for SVD, for which currently only cultivated populations are known. For SL, we sampled only one of the five known localities because this species is morphologically so obviously distinct from both *S. vespertilio* subspecies that larger samples were not needed.

### Morphological Analyses, Hand-Manipulated Interspecific Crosses, and Viability Assessments

**FIGURE 2:**
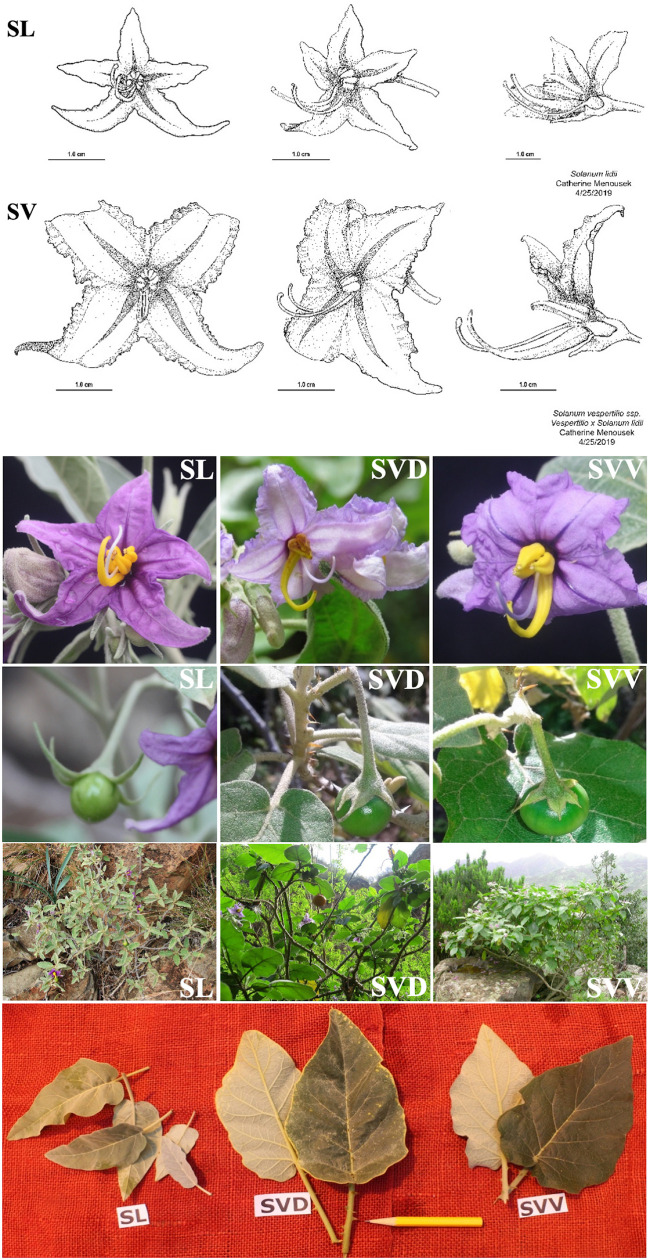
Flower structure (drawings and photos), leaves, fruits, and plants of the *Solanum* taxa endemic from the Canary Islands: *S. vespertilio* spp. *vespertilio* (SVV), *S. vespertilio* spp. *doramae* (SVD), and *S. lidii* (SL). Photos by G. Anderson, Á. Marrero, R. Jaén-Molina, and Óscar Saturno.

The plants used for the scoring or measurement of characters noted as key differences among the species (Table 2) depended on the character and depth of analysis. For instance, overall features scored for Table 2 included both field-collected data (e.g., seed/fruit) and greenhouse samples (see Figure 2). Leaf features, in particular, the length, width and length/width ratio, have been important in distinguishing the subspecies of *S. vespertilio*. Thus, leaf variation was studied more intensively in larger populations of plants grown from wild-collected seed (or the JBCVCSIC seedbank for SVD) in “common gardens” (research greenhouses at the University of Connecticut), and in hand-generated hybrids between all possible pairwise combinations of SVV, SVD, and SL. Measurements were taken on the topmost mature leaves on mature plants (i.e., several months to year-old plants), and included length of the blade from tip to the lowest point on the basal lobes of the blades (i.e., not including the petioles); the width was taken at the widest point. The length/width ratio was then calculated.

**TABLE 2:** Characterization of the three *Solanum* Canarian taxa.

Characteristic	SVV	SVD	SL
Habitat	Moist laurisilva	Moist laurisilva	Xeric rocky
Habit	Robust woody shrub	Robust woody shrub	Smaller woody shrub
Rarity	Uncommon	Extinct in wild	Rare
Andromonoecy	Strong	Strong	Weak
Autogamy	Weak (variable)	**–**	Strong
Flower size	Large	Large	Medium
Flower color	Light purple	Light purple	Darker purple
Flower parts	4	4	5
Zygomorphy	Strong	Strong	Moderate^a^
Heterandry	Strong	Strong	Strong
Enantiostyly	Weak (variable)	**–**	Moderate
Pollen distribution^b^	45%	**–**	29%
Fruit size^c^	∼2 cm	∼2 cm	∼1 cm
Number seeds per fruit^d^	50	49.6	7.1

*^a^Stamens are heteromorphic and make the androecium zygomorphic; the corolla is zygomorphic, but much less so than either S. vespertilio subspecies. ^b^Percentage in long anther. ^c^Size (diameter in mm) from Bramwell and Bramwell (1990), except for SVD which is our data. ^d^All counts from field-collected fruits.*

Artificial hybrids were generated between all three possible pairwise combinations: SVV with SVD, SVV with SL, and SVD with SL (female parents listed first in each case; see details of crossing techniques in Anderson and Levine, 1982). Seeds from successful crosses were harvested from ∼4-month-old fruits and grown for 2–4 years in the research greenhouses. We used at least 16 successful artificial hybrid “families” (i.e., the multiple seeds grown from a particular hybrid combination). For each hybrid combination, there were usually several “duplicate families” (i.e., different plants of the same taxon involved in generating that hybrid combination). The vouchers representing the parental taxa used, and hybrids generated, are in CONN (The University of Connecticut herbarium collections). We used a total of 16 hybrid families including 67 genets (separate genetic individuals) within them, from which measurements were taken (Table 3). For the parents, we used 15 genets of SVV, 8 genets of SVD and 13 genets of SL. Each parental genet was a distinct individual, but they may have been derived from seeds grown from the same fruit. Two-tailed Student’s *t*-tests were used to test for significant differences between various pairs in Table 3. Artificial hybrids were evaluated for overall growth and vigor, and then specifically for fertility by via pollen viability assayed via staining pollen grains with aniline blue in lactophenol (Anderson and Symon, 1989).

**TABLE 3:** Leaf morphology characterization of the three *Solanum* Canarian taxa.

		Genets	Length (mm)	Width (mm)	Lenght/width ratio
**SVV × SL**					
	Hybrid	27	9.7	4.3	2.3
	SVV	15	10.6*	6.5***	1.6***
	SL	13	4.6***	2.4***	1.9***
**SL × SVD**					
	Hybrid	28	10.8	5.5	2
	SL	13	4.6***	2.4***	1.9
	SVD	8	10.6	6.7***	1.6***
**SVV × SVD**					
	Hybrid	12	12.5	7.5	1.7
	VSV	15	10.6***	6.5***	1.6
	SVD	8	10.6***	6.7**	1.6

The individuals analyzed were propagated in the greenhouse. Probability (t-test with unequal variances, ***, **, and * indicate significant pairwise differences between the artificial hybrids and their respective parents at a significance level of P < 0.001, P < 0.01, and P < 0.05, respectively).

### DNA Extraction, Library Preparation, and Sequencing

Genomic DNA was extracted from freeze-dried leaves using an in-house DNA extraction protocol developed for NGS applications (Alonso et al., 2018). DNA quality and integrity were evaluated using a NanoDrop ND-1000 spectrophotometer (NanoDrop Technologies, Wilmington, DE, United States) checking 260/280 and 260/230 nm ratios and by agarose electrophoresis. DNA concentration was estimated with Qubit^®^ 2.0 Fluorometer (Thermo Fisher Scientific, Waltham, MA, United States). Samples were diluted and sent to IGA Technology Services (IGATech, Udine, Italy) for genotyping using the eggplant SPET platform, where genomic libraries were constructed and sequenced using an Illumina NextSeq 500 sequencer in single-end mode (150 bp). The eggplant SPET platform was designed using the SNP dataset (over 10.9 million SNPs) generated from the whole-genome resequencing of seven *S. melongena* and one *S. incanum* accessions (Gramazio et al., 2019) and selecting the 5,093 most reliable SNPs, 3,372 of them in CDSs and 1,721 in introns and UTRs regions, by applying stringent filter criteria and running pilot tests (Barchi et al., 2019a).

### Reads Processing, Mapping, and SNP Calling

After sequencing, the base calling and demultiplexing steps were performed using a standard Illumina pipeline, while read quality check and adapter trimming were accomplished using ERNE (Del Fabbro et al., 2013) and Cutadapt (Martin, 2011) software, respectively. Subsequently, reads were mapped to the reference genome “67/3” (Barchi et al., 2019b) using BWA-MEM aligner (Li, 2013) with default parameters and only uniquely aligned reads (i.e., reads with a mapping quality > 10) were selected). Variant calling was performed following the Germline short variant discovery (SNPs + Indels) workflow^2^ using the GATK 4.0 software (Depristo et al., 2011). Specifically, the variants were called per-sample by the HaplotypeCaller (Poplin et al., 2017) to generate an intermediate GVCF (Genomic Variant Call Format) file for each sample. The GVCFs files from multiple samples were consolidated into a GenomicsDB datastore to improve scalability and gathered together to the GenotypeGVCFs tool for a cohort-wide joint-called genotyping analysis. Reduction of the number of false positives was performed with the SelectVariants and VariantFiltration tools to apply stringent filter criteria (QD < 2.0, MQ < 40.0 and MQRankSum < −12.5). To select only highly reliable SNPs, we applied stringent criteria to the VCF file using VCFtools (version 0.1.15) (Danecek et al., 2011) with the following parameters: min-meanDP 30, minDP 30, max-missing 0.95 and maf 0.02. After checking the reliability and efficiency of the SPET technique (i.e., no polymorphisms between the two technical replicates of a sample), we decided to remove duplicates for the subsequent analyses.

### Molecular Data Analysis

To analyze and manipulate the high-throughput genotype data, the VCF file was uploaded to R (Ihaka and Gentleman, 1996) using the vcfR package (Knaus and Grünwald, 2017) and the data transformed to genlight and genind objects using the function vcfR2genlight of the R package Adegenet (Jombart, 2008). Basic statistics were estimated using the VCFtools software (Danecek et al., 2011) and the function basic.stats of the R package hierFstat (Goudet, 2005) based on the analysis of gene diversity proposed by Nei (1973). Confidence intervals for population−specific *F*_is_ were estimated using the boot.ppfis function in hierfstat by performing 10,000 bootstrap replicates over loci. Pairwise *F*_st_ values among populations were calculated for populations with four or more individuals (*n* ≥ 4) (Willing et al., 2012). To clarify the complex relationships among the accessions from the three endemic Canarian *Solanum* taxa and provide insight into their population structure, we carried out complementary analyses. First, a hierarchical clustering based on pairwise similarities was performed to assess the genetic relationships among the 74 accessions. A consensus UPGMA (unweighted pair group method with arithmetic mean) dendrogram based on Nei (1972, 1978) genetic distance was obtained using the function about of the R package poppr (Kamvar et al., 2014). Branches of the dendrogram were tested with bootstrap support for Bruvo’s distance (Bruvo et al., 2004) with 1,000 replications. The dendrogram was graphically plotted using the function plot.phylo of the R package ape (Paradis and Schliep, 2018). To confirm the reliability of the UPGMA dendrogram, a SVDQuartets analysis was performed in PAUP^∗^ program (Swofford, 2003). The SVDquartets method (Chifman and Kubatko, 2014, 2015) is a coalescent-aware phylogenetic method based on nucleotide sequence data that can identify the correct species tree in the face of deep coalescence leading to incomplete lineage sorting (ILS). ILS can lead to artifacts in standard methods/models when there are short branches anywhere in the tree and effective population sizes are large enough that sister lineages do not coalesce before (looking backwards in time) their common ancestor. To infer the population structure and detect additional sub-populations, a fastSTRUCTURE analysis, which is based on a variational Bayesian approach, was performed (Raj et al., 2014). To convert the data from the VCF file to the fastSTRUCTURE format, the software PGDSpider (Lischer and Excoffier, 2012) was used. A principal component analysis (PCA) was performed using the function glPCA of the package Adegenet (Jombart, 2008), and represented graphically using the package ggplot2 (Wickham, 2016).

### Molecular Data Access

The sequence data have been deposited into the NCBI Short Read Archive under the Bioproject identifier PRJNA556343. Raw reads of each accession are deposited under the accession numbers from SRX6649376 to SRX6649471. VCF files with the corresponding variants identified are available upon request to the corresponding author.

## Results

### Analysis of Morphology and Pollen Stainability

Both subspecies of *S. vespertilio* are distinct from *S. lidii* in terms of leaf length and width, but less distinct in terms of the length/width ratio (Table 3). Analyses of the artificial hybrids resulting from hand-generated crosses between SL and either parental subspecies shows that they are mostly distinct as well, with some features (e.g., leaf length and width) similar to one species or the other, but with some hybrids manifesting intermediate characters or heterosis. The hybrids between the two subspecies of *S. vespertilio* are also distinct in leaf length and width, but not in the length/width ratio. All three taxa and all the hybrid combinations were, on average, fully fertile, with pollen staining percentages averaging from 80 to 95%. Thus, none of the taxa, nor the artificial hybrids between them, manifested sterility.

### High-Throughput Sequencing and Mapping

After the sequencing, cleaning and trimming steps, over 5 billion bp were generated in the 74 accessions used in this study, which correspond to over 33 million clean reads of 150 bp (Supplementary Data S1). Due to the different quality of the plant material (some samples were stored for a longer time than others), the number of sequenced reads per sample varied considerably among the accessions with an average of 446,472 clean reads per sample (range from 124,841 of SL_TE_9 and to 1,801,500 of SVV_CO_2). Of those reads, on average, 95.3% were mapped onto the eggplant reference genome “67/3” (Barchi et al., 2019b). The average of the mean accession coverage considering the target regions in each accession was 78.8 reads, ranging from 22.0 for sample (SL_TE_9) to 316.8 reads for sample (SVV_CO_2); thus, deep and reliable large enough for most of the bases sequenced to be mapped.

### SNP Calling and Population Structure Statistics

A total of 6,504 SNPs were identified using the 5,093 probes of the eggplant SPET platform (Table 4). The distribution of the SNPs identified along the chromosomes was uneven and ranged from 336 of chromosome 11–951 of chromosome 1 with an average of 542 SNPs per chromosome. Surprisingly, there were only 284 targeted SNPs identified (i.e., SNPs selected for the SPET platform), around 5.6% of the total set, while there were 6,220 untargeted SNPs (i.e., novel SNPs identified in the surroundings of the targeted SNPs), almost 22-fold more than the targeted SNPs. On the other hand, even though the average of the mean probe coverage was 86.9 reads, some of the probes showed low coverage (Supplementary Data S2), so that stringent criteria were applied to retain only the most reliable SNPs. After filtering, a total of 1,421 high-quality SNPs were selected and used for the subsequent analyses. Only 34 SNPs were targeted SNPs (0.67% of the SPET set), while there were 1,387 novel SNPs, over 40-fold greater than the targeted SNPs (Table 4).

**TABLE 4:** SNP distribution per chromosome identified among the 74 accessions from the three *Solanum* Canarian taxa.

	Chr. 1	Chr. 2	Chr. 3	Chr. 4	Chr. 5	Chr. 6	Chr. 7	Chr. 8	Chr. 9	Chr. 10	Chr. 11	Chr. 12	Average	Total
**Whole SNP set**	951	339	606	479	412	603	618	465	557	693	336	445	542.0	6,504
*Targeted SNPs*	49	13	21	16	24	24	21	26	31	26	12	21	23.7	284
*Untargeted SNPs*	902	326	585	463	388	579	597	439	526	667	324	424	518.3	6,220
**Filtered SNP set**	226	56	147	100	81	145	118	83	133	152	84	96	118.4	1,421
*Targeted SNPs*	6	1	4	2	4	1	0	3	5	3	3	2	2.8	34
*Untargeted SNPs*	220	55	143	98	77	144	118	80	128	149	81	94	115.6	1,387

Most of the SNPs identified were biallelic and only 13 presented more than two alleles. The average of the reference allele frequency (i.e., the same as the reference genome) was 0.782 with a wide range from 0.014 to 0.993 (Supplementary Data S3). Overall, the number of heterozygous SNPs was low, as reflected in the low average value for the observed heterozygosity per locus (*Ho*; 0.113), in the average within-population gene diversity per locus (*Hs*; 0.110) and in the average total population gene diversity per locus (*Ht*; 0.198), respectively. As well, the average inbreeding coefficient (*F*_is_) per locus was 0.085. However, the average fixation index per locus (*F*_st_) was 0.331, indicating moderate to a high level of differentiation due to the members of some populations carrying unique alleles compared to the rest. On the other hand, the average gene diversity (*D*_st_) per locus among subpopulations was low (*D_st_* = 0.088), and ranged from 0 to 0.403, similar to the average measure of population differentiation (*D*_est_) per locus (*D_est_* = 0.116), which ranged from 0 to 0.510. Population SL_TE showed the highest average observed heterozygosity (*Ho*) per locus with a value of 0.230, followed by populations SVV_BR (0.134), SVV_AR (0.128), and SVV_ME (0.121); the lowest value was observed for SVD_O (0.039) (Table 5 and Supplementary Data S4). A similar pattern was found for gene diversity, where SL_TE showed the highest average value per locus (0.208) and population SVD_O the lowest (0.022). For the average *F*_is_, populations SVV_BO and SVV_ME showed the only average positive values (0.170 and 0.090, respectively), while population SVD_O presented the most negative average values with −0.798. Individual values for all the accessions for *Ho*, *He*, and *F*_is_ are reported in Supplementary Data S5. The estimated *F*_st_ values between pairwise populations displayed low values among SVV populations, ranging from 0.106 between SVV_ME and SVV_BR to 0.296 between SVV_AR and SVV_CO (Table 6). The values comparing the geographically most separate SVV_CO (from Teno, on Tenerife) with other SVV populations were consistently the highest. *F*_st_ values between SVD_O and SVV populations were even higher, apart from the comparison with SVV_BO (0.109). Not surprisingly, the SL_TE pairwise values were generally uniformly higher among all the comparisons, interestingly the highest for the comparison with SVV_CO (0.431).

**TABLE 5:** Population structure statistics for eight populations from the three *Solanum* Canarian taxa. Population codes as in Table 1.

	SVV_BR	SVV_BO	SVV_AR	SVV_ME	SVV_CO	SVD_O	SVD_I	SL_TE
Observed heterozygosity	0.134	0.104	0.128	0.121	0.078	0.039	0.072	0.230
Gene diversity	0.129	0.114	0.111	0.130	0.070	0.022	NA	0.208
Inbreeding coefficient (*F*_IS_)	−0.013	0.170**	−0.126***	0.090**	−0.056**	−0.798***	NA	−0.079***

***, **, and *, ns indicate significant difference at p-values < 0.001, < 0.01, and < 0.05, respectively.

**TABLE 6:** Pairwise *Fst* estimates between *Solanum vespertilio* subsp. *vespertilio*, *S. vespertilio* subsp. *doramae*, and *S. lidii* populations with number of individuals (*n* ≥ 4).

	SVV_BR	SVV_BO	SVV_AR	SVV_ME	SVV_CO	SVD_O
SVV_BO	0.125	–				
SVV_AR	0.127	0.152	–			
SVV_ME	0.106	0.141	0.169	–		
SVV_CO	0.267	0.283	0.296	0.257	–	
SVD_O	0.294	0.109	0.387	0.285	0.408	–
SL_TE	0.335	0.357	0.349	0.346	0.431	0.388

### Private and Discriminant SNPs

One of the main aims of this study, apart from establishing genetic relationships among the accessions assessed, was to provide genetic tools to easily discriminate the three taxa. As expected, given the larger morphological distinction, the largest set of private SNPs (i.e., SNPs specific to a particular taxon or population) was found for SL with 55 SNPs that discriminate it from the two subspecies of *S. vespertilio* (Supplementary Data S6). Private SNPs for SL were found to be distributed across all chromosomes except in chromosome 5, although the number of SNPs in chromosomes carrying private SNPs varied considerably, ranging from chromosome 1 with 17 SNPs to chromosome 11 with just one SNP. However, not all the private SNP loci provide the same discriminatory power because some of them shared SNP alleles with some accessions of the two subspecies of *S. vespertilio*. The most informative and reliable private SNPs, i.e., those represented by the same homozygous alleles for all the individuals of SL (e.g., A/A), and different homozygous alleles for the rest of the accessions of the two subspecies of *S. vespertilio* (e.g., C/C), were selected to create a subset called “Core private SNPs.” This subset presented 36 SNPs with five SNPs on chromosomes 3, 7, 10, and 12, four SNPs on chromosomes 1 and 6, three SNPs on chromosomes 4, two SNPs in 2 and 8 and just one SNP on chromosome 11, while chromosomes 5 and 9 did not bear any SNPs. Some of the private SNPs in the Supplementary Data S6, like other SNPs in the full set of SNPs, can be considered as potential haplotypes because they are physically very close and probably are tightly linked and inherited together. However, all the analyses were conducted using SNPs as a single information unit and not as haplotypes. For the two subspecies of *S. vespertilio*, only one private SNP was found for SVV and none for SVD. However, for SVD, when the accession (SVD_I_1) is removed from the analyses, seven private SNPs were identified between SVV and SVD, although none of them was specific and universal and therefore did not meet the requirement for being included in the “Core private SNPs” subset.

### Population Structure and Genetic Relationships Analysis

**FIGURE 3:**
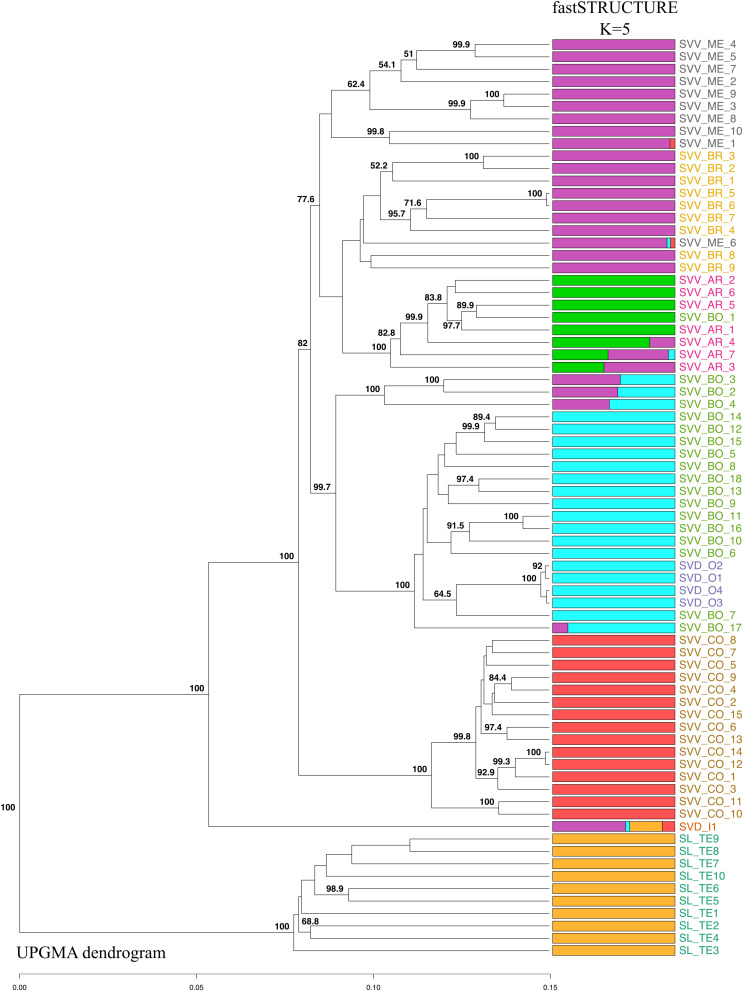
Genetic population structure of the 74 accessions from the three Canarian endemic *Solanum* taxa according to an UPGMA-based dendrogram and a fastSTRUCTURE (*K* = 5) analysis with 1,421 SNPs. Bootstrap values greater than 50%, based on 1,000 replications, are indicated in percentage at the corresponding nodes. Each accession is represented by a horizontal bar that is partitioned into colored segments corresponding the estimated membership of an accession to one of the five specific inferred clusters. Population codes as in Table 1 and colors as in Figure 1.

The UPGMA-based dendrogram obtained for 74 accessions revealed a sister basal clustering of all SL individuals, although the relationships among them were not always well supported by bootstrap values (Figure 3). Interestingly, the SVD accession from Moya (SVD_I_1) was placed outside of and as basal sister to the rest of the accessions of SVD, and close to the SL accessions. The fastSTRUCTURE analysis (Figure 3) also points to the connection of this SVD to SL, in that it is the only SVD that shows a genetic admixture with SL. The remainder of the SVD accessions (from Teror, SVD_O1-4) nested in the middle of the SVV accessions from the northeastern populations on Tenerife (the Anaga peninsula). The SVV samples from Teno clustered together in a separate branch from all the accessions of SVV collected in Anaga. This Teno group is as distinct from the rest of the subspecies SVV as the single Moya accession of SVD (SVD_I_1) is from all the other SVV (and SVD) accessions. Except for a few samples that were intermingled in other groups, the four populations from Anaga constituted distinct individual clusters.

**FIGURE 4:**
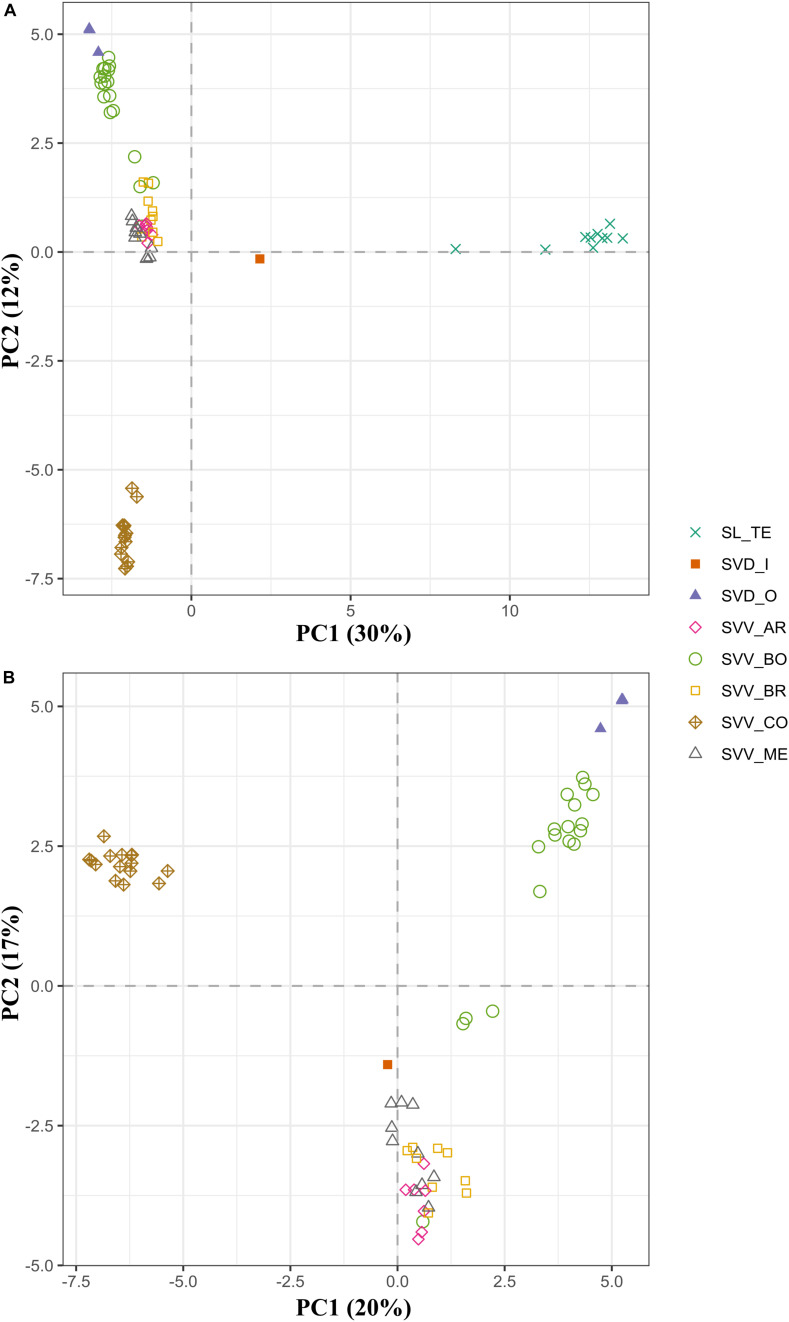
Principal coordinates analysis (PCA) similarities based on the whole set of SNPs (1,421) for the 74 accessions from the three endemic *Solanum* taxa in the Canary Islands **(A)** and on partitioned data without *S. lidii* accessions **(B)**. The first and second principal coordinates are displayed. Population codes as in Table 1 and colors as in Figure 1.

Although there were some minor rearrangements in branch disposition, the two different methods for estimating species trees (UPGMA and SVDquartets), gave the same topology of accessions, confirming the results of the UPGMA analysis that we show herein, and suggesting that there are no significant artifactual groupings due to deep coalescence (Supplementary Data S7). The fastSTRUCTURE analysis suggested that the optimal number of genetic clusters was obtained at *K* = 5. Only two clusters (red and yellow) consisted mainly of the individuals from a single population (or species) (Figure 3). In fact, individuals from SL and those from Teno SVV_CO showed perfect intrapopulation uniformity with apparently no indications of hybridization or migration from other groups. This pattern, where all individuals of one population were linked in one part of the diagram, was also observed for SVD_O and SVV_BR, although, in this instance, they shared clusters with other populations SVV_BO in the case of SVD_O and SVV_ME for SVV_BR. This pattern suggests that some event led to a sharing of alleles and genetic structure. However, while in the case of SVV_ME for SVV_BR geographical proximity might have facilitated the contact, in the case of SVV_BO (from Tenerife) and SVD_O (from Gran Canaria) the two populations are from islands separated by 80 km. On the other hand, SVV_BO and SVV_AR showed less uniformity with fragments from three different clusters. Overall, the multivariate PCA analysis bolstered the results of the dendrograms, although some groups were nested (Figure 4A). The first principal coordinate, which explained 30% of the genetic variation, clearly separated the SL accessions and the SVD_I_1 accession from the rest, while the second coordinate, which accounted for 12% of the variation, separated the SVV Teno accessions from the rest of SVV accessions from Tenerife (notably also including all the SVD accessions except the single SVD Moya accession (SVD_I_1). To enhance resolution among the SVV groups, an additional PCA analysis was performed on data that did not include the SL accessions (Figure 4B). The first axis, which accounted for 20% of the genetic variation, divided the accessions from Teno from the rest of the *S. vespertilio* populations, while the second axis, constituting 17% of the genetic variation, strongly separated the SVD_O (Teror) accessions from the SVD_I_1 (Moya) accession. Furthermore, this second coordinate separated almost all the SVV accessions from Lomo de Las Bodegas SVV_BO from the rest of the Anaga SVV populations SVV_BR, SVV_AR and SVV_ME. The latter showed a strong admixture pattern.

## Discussion

### High-Throughput Genotyping SPET Platform

The eggplant SPET platform has been reliably applied to species closely related to eggplant (Barchi et al., 2019a), and this investigation shows that it is transferable to *S. lidii* and *S. vespertilio*, two rare Canary Island endemics much more distantly related to the eggplant. Therefore, this study provides additional proof of the reliability of this technology as well as of its wide cross-species transferability. This new genotyping solution combines the benefits of (a) complexity reduction (e.g., high-throughput and cost-effective), (b) high-density DNA arrays (e.g., targeted and reproducible), and (c) discovery of novel polymorphisms in addition to the target SNPs in regions of interest, thus allowing high-transferability across species (Scaglione et al., 2019). The number of SNPs identified in this investigation (6,504) was considerably higher than the number of the probes designed (5,093). Furthermore, only 284 polymorphisms were targeted SNPs versus the 6,220 novel SNPs detected in the 74 accessions. Using single-end sequencing of 150 bp, after removing 40 bp of the probe, up to 110 bp per read have been mapped onto the reference genome (Barchi et al., 2019b); this allowed us to screen the flanking regions of the targeted SNP for novel and private SNPs. In addition, this strategy allows the detection of potential haplotypes among populations as well as SNPs in linkage disequilibrium (Scaglione et al., 2019). An additional benefit of the high reproducibility of SPET is that it could allow data to be merged from independent studies that use the SPET platform (with the same configuration of probes) to facilitate much broader-based phylogenetic meta-analyses. Ultimately, novel analytical technologies like SPET (Barchi et al., 2019a; Scaglione et al., 2019), provide great strength in a range of studies, including those like this where our goal is to understand island colonization patterns, evolution and suggest conservation strategies for rare taxa.

### Relationships Among Taxa and Tentative Hypotheses on Their Evolution on Islands

*Solanum lidii* and *S. vespertilio* are clearly separated by morphology and molecular data. Interestingly, the hybrids between them are intermediate and fertile. Särkinen et al. (2013) synthesis has given high support to that the species *S. lidii* and *S. vespertilio* are derived from common ancestors of the African *Solanum* subgenus *Leptostemon* taxa. Also, Särkinen et al. (2013) suggest that the common ancestor of the Canarian *Solanum* taxa split from African lineages about 1.68 Myr (though there is a wide standard error associated with the age estimates, see Supplementary Material). Assuming that this estimate represents the stem age of *Solanum* in the Canaries (i.e., the colonization of the archipelago by the ancestor of the current endemics), it would mean that these taxa are recent endemics on Gran Canaria and Tenerife, in spite of the fact that these two large islands are among the older in the archipelago (they emerged 15 and 12 Myr respectively; Van Den Bogaard, 2013).

The date of first colonization of the Canaries by *Solanum* is unknown, but it may have been even later, closer to the suggested date at which *S. lidii* and *S. vespertilio* became distinct lineages (i.e., 0.93 Myr; Särkinen et al., 2013). From present data, we cannot be sure whether that common ancestor migrated to the Canaries or it split in Africa yielding the ancestral forms of *S. lidii* and *S. vespertilio* prior to colonization. The cladogram (Figure 3) also cannot answer the question of whether the common ancestor was more similar to what we now recognize as *S. lidii* or *S. vespertilio*. However, the ecology and habitat preference of the proposed extant closest relatives (African species: *S. capense, S. tomentosum*, and *S. humile*; Särkinen et al., 2013) suggest a hypothesis: the continental African taxa occupy similar habitats to that of *S. lidii* that are drier, often disturbed, open woodland, sandy or rocky places (Vorontsova and Knapp, 2016)^3^. If the common ancestor to the Canarian taxa was similar, then the descendants of *S. lidii* could have grown first in these more xeric habitats on both Gran Canaria and Tenerife, and then radiated into the more mesic-moist scrub land yielding the selection that gave the larger-leaved, more robust, less-tomentose *vespertilio-*like plants. Similar patterns are reported for the colonization of Hawaii by Baldwin and Warren (2010). However, the lack of any evidence for *S. lidii* on Tenerife at any time argues against it occurring there.

*Solanum vespertilio* may have split with *S. lidii* from the common African ancestor and adapted independently to the more mesic habitats on both islands. If the split from the common ancestor that yielded the two taxa occurred after Canarian colonization, this would suggest that *S. vespertilio* occurred first on Gran Canaria and then underwent short-distance dispersal (∼80 km) to Tenerife, where it proliferated in the more common moist habitats. In this scenario, *S. vespertilio subsp. doramae* (today virtually extinct-in-the-wild) can be construed as one remnant of that split and further radiation into what are today increasingly uncommon undisturbed moist habitats on Gran Canaria. The position in the tree (Figure 3) of SVD_I (a descendant of individuals of SVD collected from the wild in Moya) supports this hypothesis, in that it is intermediate between *S. lidii* and the other SVV and SVD accessions. The molecular profile of the other SVD accessions firmly embeds them within the Tenerife SVV accessions. Consistent with data from morphological analyses and hybridization studies, these genotypically and morphologically indistinct SVD accessions are compatible with the conclusion that there may have been contemporary natural hybridization between typical *S. vespertilio* subsp. *vespertilio* and the Gran Canarian *S. vespertilio* subsp. *doramae*. Alternatively, the original samples may have already undergone rounds of hybridization (see the “conservation implications” section below).

The SVV accessions are distributed in Anaga and Teno massifs (Figure 1), with estimated geological ages of respectively 3.9–4.9 Myr and 5.6–6.2 Myr, and therefore much older than the central massif of Tenerife (Carracedo and Day, 2002). At least 13 endemic plant species share with SVV this disjunct distribution between the West (Teno) and East (Anaga) of Tenerife (Moya et al., 2004; Trusty et al., 2005; Mairal et al., 2015). The geographical gap between these two ends of Tenerife is clearly reflected in the tree (Figure 3) and in the PCA (Figure 4). This pattern of genotype distribution is similar to those reported in phylogeographic investigations of the Canarian flora and has been detected (to different extents) in either widespread or more narrowly distributed Canarian plant endemics belonging to disparate lineages, like *Canarina canariensis* (Campanulaceae, Mairal et al., 2015), *Dorycnium broussonetii* (Fabaceae, Jaén-Molina et al., 2015), *Ruta pinnata* (Rutaceae, Soto and Jaén-Molina, unpublished data), or *Navaea phoenicea* (García et al., 2009).

Not all the lineages with an Anaga-Teno distribution are old. Northern Tenerife has been subjected to a number of geologic events such as volcanic landslides and avalanches in different periods of time from 2.2 Ma (Tigaiga landslide) to 170 ka (formation of Las Cañadas Caldera) (Ancochea et al., 1990; Watts and Masson, 1995; Ablay and Hürlimann, 2000) that likely have caused recent disruptions of gene flow in taxa that formerly constituted a continuum of populations. For instance, the populations of *Bystropogon* (Trusty et al., 2005), and some populations of *Micromeria* (Puppo et al., 2015) or *Canarina canariensis* (Mairal et al., 2015), presently distributed in Teno and Anaga may be the remnants of a larger ancestral distribution area affected by these volcanic events. In the case of *Navaea phoenicea* (Malvaceae), a palaeo-endemic with a similar distribution to SVV, González De (2016) finds modest levels of gene flow mediated by pollinators among Anaga and Teno. Our genetic data in *Solanum* do not provide support for current gene flow between these widely separated areas, so that the separation between the distribution areas in Teno and Anaga might well be more ancient than the reported upheavals.

These putative barriers to gene flow may also mean that the two geographical elements of *S*. *vespertilio* on Tenerife may constitute separate subspecies, as genetically differentiated as *S. vespertilio* on Gran Canaria (SVD) is from SVV on Tenerife. The fact that the individuals from Teno show perfect intrapopulation uniformity with no indications of hybridization or migration from other groups in the fastSTRUCTURE analysis (Figure 3) provides further support of the possibility that this population originated from a founder event independent of the SVV in Anaga.

The pattern of relationships suggested by the SNP data (Figure 3; i.e., the nesting of the samples of SVD_O from Gran Canaria within the SVV Anaga populations) implies a closer relationship between the SVV from the Teno region of Tenerife and SVD from Gran Canaria. In the context of the currently accepted taxonomy of *S. vespertilio*, our results and the geographical closeness between Anaga and Gran Canaria suggest that dispersal from Anaga to Gran Canaria could have occurred much later than the interruption of gene flow between the populations of SVV from Anaga and Teno. The facts that (a) the area of Anaga has been ecologically (if not geologically) stable historically, and (b) it reportedly acted as a source of genetic diversity for other regions in the archipelago (Mairal et al., 2015), provide support to this first hypothesis. Our morphological data support as well the hypothesis that more recent natural hybridization between introduced SVV and some of the Gran Canarian SVD.

### Taxonomic Implications

The experimental analyses of a large sample of common garden-grown plants and the molecular data confirm that the two species, *S. lidii* and *S. vespertilio*, are clearly distinct. The common garden studies show that the subspecies of *S. vespertilio* are morphologically very similar. Interestingly, the results of the SNP genotypic analyses suggest great populational variation, and the possibility of a complex colonization pattern involving SVV, SVD, and SL. Combined with the experimental hybridization studies and morphology, these data bolster the hypothesis of a recent hybridization between SVV and SVD. As noted, hybrids between the subspecies are easy to obtain in the greenhouse and they are fertile. Morphologically, the subspecies are, as is often the case, not very distinct: the morphology of the hybrids is either intermediate or a mixture of greater similarity to one parent than to the other. However, the high fertility of hybrids between the unquestionably distinct *S. lidii* and *S. vespertilio* also suggests that all three of the currently recognized Canarian native *Solanum* taxa are genetically very closely related. Thus, although infertility studies of hybrids can sometimes help evaluate species distinction (e.g., Anderson, 1977, 1979), in other instances the combination of genomes in hybrids is not inhibited by pre- or post-zygotic barriers, and species boundaries are presumably more ecologically or geographically based (Whalen and Anderson, 1981; Plazas et al., 2016). Furthermore, although there may be morphological and ecological differences between taxa, these do not necessarily imply more wholesale genome differences that might be manifest in F_1_ hybrid sterility.

In this case, artificial hybridization tells us that all three taxa are quite similar for a number of key genotypic characters, i.e., hybrids are not separated by genetic factors that are integral to the production of reproductive cells. Thus, not surprisingly, the subspecies are also inter-compatible, yielding hybrids with high fertility, an outcome not unprecedented in Angiosperms, not even in the genus *Solanum* (Plazas et al., 2016).

The lack of a suite of consistently expressed morphological characters distinguishing the subspecies is problematic (Table 2). However, the geographic and genotypic distinction of one of the few remaining representatives of *S. vespertilio* ssp. *doramae* (SVD_I_1 from Moya), and the nesting of other accessions of SVD fully interspersed within the collections of SVV (Figure 3) suggests that many of the presumed SVD samples (for instance those planted in Osorio) may constitute recent hybrids between SVV and SVD, raising important points for conservation programs that are considered further in the section below.

As noted above, the SNP genotypic data highlight a very distinct genotypic population of *S. vespertilio* from Teno (Bco. Los Cochinos, Los Silos), a more arid region in the northwest of Tenerife. The Teno samples of SVV are notably different from all other Tenerife accessions. Thus, these data perhaps reflect the geological and geographical distinction of the Teno area as a paleo-island (Carracedo and Day, 2002). A thorough morphological analysis is being carried out to confirm whether there are sufficient and consistent morphological characters to justify a new taxon within *S. vespertilio* (Marrero et al., in prep.).

### Conservation Implications

The rareness and vulnerability of all the taxa considered, especially the two from Gran Canaria, make this study extremely important for designing effective conservation strategies and restoration programs that are based on the full understanding of the relationships, genotypic diversity, ecological requirements and reproductive biology (including pollination biology) of the most threatened taxa. For instance, there is an interesting paradox in the context of mating systems (Neal and Anderson, 2005) as applied to conservation. All three currently recognized taxa are capable of autogamy, though at a relatively low rate. Interestingly, the entity with the highest rate of autogamous fruit set in experimental settings (*S. lidii*), has the lowest fixation index (*F*_is_), implying low self-breeding, in spite of starkly declining population sizes.

Our results show average *F*_is_ values (range between 0.301 and 0.587) for the larger and more widespread *S. vespertilio* (SVV) populations. These values make sense in terms of the reproductive biology and population biology of SVV as we understand it. However, they do not for *S. lidii* (SL), where *F*_is_ is negative (-0.204), indicating an excess of heterozygotes. This is uncommon and could be caused by either: (a) previous hybridization between genetically very different SL populations that have not yet reached Hardy-Weinberg equilibrium, or (b) natural selection for highly heterozygous individuals. However, regardless of the underlying cause, these perspectives are important for effective conservation design. Hybridization of SVV, SVD, or SL with other *Solanum* species occurring in the Canary islands, including the cultivated *S. melongena* L., that might endanger the genetic integrity of these Canarian endemics, is unlikely given the different habitats and phylogenetic distance with other *Solanum species* occurring in the Canary islands (Vorontsova and Knapp, 2016), the small size of the endemic populations, low success after artificial hybridization with cultivated eggplant (Kouassi et al., 2016), and the potential sterility of interspecific hybrids. Low *F*_st_ values for the comparisons of SVV with SVD indicates low genetic differentiation between these two subspecies, in spite of their distribution on separate islands. These lower values may suggest genetic flow between SVV and SVD, which may endanger the genetic integrity of the highly vulnerable SVD. To the contrary, the higher *F*_st_ values for comparisons of the geographically distinct SVV from Teno suggests that this entity has been less subject to hybridization with other SVV, SVD, or SL populations. Thus, the proposed subspecies (Marrero, in prep.) is likely to be more stable. In this way, for the distinctive Gran Canarian genotypes (SVD) to be maintained, conservation plans must limit the growth of introduced SVV near SVD. Indeed, the nesting of the presumably “pure” SVD individuals sampled at Finca de Osorio (Gran Canaria) within the SVV samples from Las Bodegas (Tenerife) (see Figure 3) indicates that these SVD samples may, in fact, be the result of hybridization with SVV (hybrids may have been easily achieved given the lack of intrinsic breeding barriers, and the cultivation at Osorio of several collections of SVV from Tenerife). Another explanation that cannot be ruled out would be that the plants introduced in Osorio SVD_O were originally subsp. *vespertilio* from Tenerife and may have been misidentified as the morphologically similar subsp. *doramae.* These issues highlight the necessity of careful identification and tracking of material introduced into conservation nurseries, and the essential value of molecular tools to achieve this objective, especially in cases of close morphological similarity. Given the results of the molecular analysis, and the forthcoming treatment of the subspecific ranks of *S. vespertilio* (Marrero et al., in prep.), we suggest that the SVD_I source, which is the most differentiated from SVV, might be considered for conservation-restoration efforts. Similarly, independently of whether the two areas of distribution of *S. vespertilio* subsp. v*espertilio* on the palaeo-islands of Tenerife (Anaga and Teno) represent distinct subspecies or not, their clear genetic differentiation indicates that *in situ* conservation efforts should avoid genetic mixing between them because the populations could be the result of two different evolutionary histories. At the minimum, the ravine in Teno (“Los Cochinos”) harbors a genotypically and ecologically distinct population that makes it a biodiversity micro-reserve, a refugium perhaps, of *Solanum* diversity (similar to conclusions by others, e.g., Mairal et al., 2015; Puppo et al., 2016; Soto et al., in prep.). Thus, new populations should be established with local genetic stock, under strict genetic traceability, and in ecologically similar nearby areas (Marrero-Gómez et al., 2010). And, obviously, irrespective of plant genotypes, removing competing invasive plants and eliminating herbivores like feral goats (Campbell and Donlan, 2005) or rabbits (Stuessy et al., 2017b, c) from islands are powerful conservation tools to prevent extinction and allow recovery of ecosystems.

Finally, as with most considerations of biodiversity, it is difficult to assess how future climate scenarios may affect the endemic populations we studied. However the current cloud forests of the Canaries generated by trade winds, crucial for the water balance in many of the natural habitats for *S. vespertilio* in particular (Kunkel, 1975b; Bramwell and Bramwell, 1990; Marrero-Gómez et al., 2010), have been predicted to decline in the forthcoming decades due to likely climate change (Sperling et al., 2004; Otto et al., 2017; Mairal et al., 2018). Given these vulnerabilities, the seed stocks of these *Solanum* kept at the seed bank of the JBCVCSIC should be reinforced to ensure success in future restoration efforts.

## Conclusion

A broad combination of approaches employed in this study of the endemic *Solanum* of the Canary Islands indicates that complex morphological and molecular variation is distributed among these taxa. Although they are still capable of sexual reproduction, suitable habitats are diminishing and populations are seriously declining. To successfully protect and restore native populations of these *Solanum* and other oceanic island endemics (Stuessy et al., 2017b), the biology of the plants and the distribution of diversity among them must be fully understood.

Our results showed that the three taxa display clear morphological differences, but they are fully cross-compatible, which may endanger their genetic integrity. The novel SNPs and SPET technology we utilized provides a powerful tool that, together with other multidisciplinary data generated, build the comprehensive foundation necessary for the successful *ex situ* management and *in situ* restoration of the Canarian *Solanum* and other island taxa. In this context, despite the small population sizes, we have found low levels for the inbreeding coefficient and a low genetic differentiation between SVV and SVD, suggesting that genetic flow that may compromise the genetic integrity of SVD may have taken place. Our results on relationships and genetic differentiation also suggest that an additional taxonomic entity, in addition to SVV and SVD, may exist within *S. vespertilio*.

Importantly, the different complementary approaches that we applied provide solid foundations for the conservation strategies of *S. vespertilio* and *S. lidii*. We consider that dialogue among scientists and various stakeholders is necessary to implement recovery plans that reduce direct threats such as land development, feral herbivores, invasive species, and genetic contamination on these endangered Canarian endemisms.

## Data Availability Statement

The datasets generated for this study can be found in the sequence data have been deposited into the NCBI Short Read Archive under the Bioproject identifier PRJNA556343. Raw reads of each accession are deposited under the accession numbers from SRX6649376 to SRX6649471.

## Author Contributions

PG and RJ-M: data curation, project administration, and writing – original draft preparation. PG, RJ-M, SV, and GA: formal analysis. PG, RJ-M, SV, JP, ÁM, JC-C, and GA: methodology, writing – review and editing, and data visualization. JP and JC-C: resources. JP, JC-C, and GA: supervision and research design and funding management.

## Conflict of Interest

The authors declare that the research was conducted in the absence of any commercial or financial relationships that could be construed as a potential conflict of interest.

## Footnotes

1 https://www.miteco.gob.es/es/biodiversidad/legislacion/leg-espanola-conservacion-especies.aspx

2 https://software.broadinstitute.org/gatk/best-practices/workflow?id=11145

3 http://solanaceaesource.org/

## Supplementary Material

DATA S1 Statistics of sequencing and mapping onto the reference genome “67/3” (Barchi et al., 2019b).

DATA S2 Mean probe coverage of the high-quality reads using the eggplant SPET platform (Barchi et al., 2019a).

DATA S3 SNP and population structure statistics.

DATA S4 Population structure statistics for the eight populations from the three *Solanum* Canarian taxa.

DATA S5 Population structure statistics for the accessions from the three *Solanum* Canarian taxa used in this study.

DATA S6 Set of private SNPs and core private SNPs for SL, SVV, and SVD.

DATA S7 SVDquartets dendrogram.
