# Supplementary figures and images for: Fostering Conservation via an Integrated Use of Conventional Approaches and High-Throughput SPET Genotyping: A Case Study Using the Endangered Canarian Endemics Solanum lidii and S. vespertilio (Solanaceae)
Source: Front Plant Sci. 2020 Jul 10;11:757. doi: 10.3389/fpls.2020.00757 (PMC7381301; doi:10.3389/fpls.2020.00757)

# SVDquartets

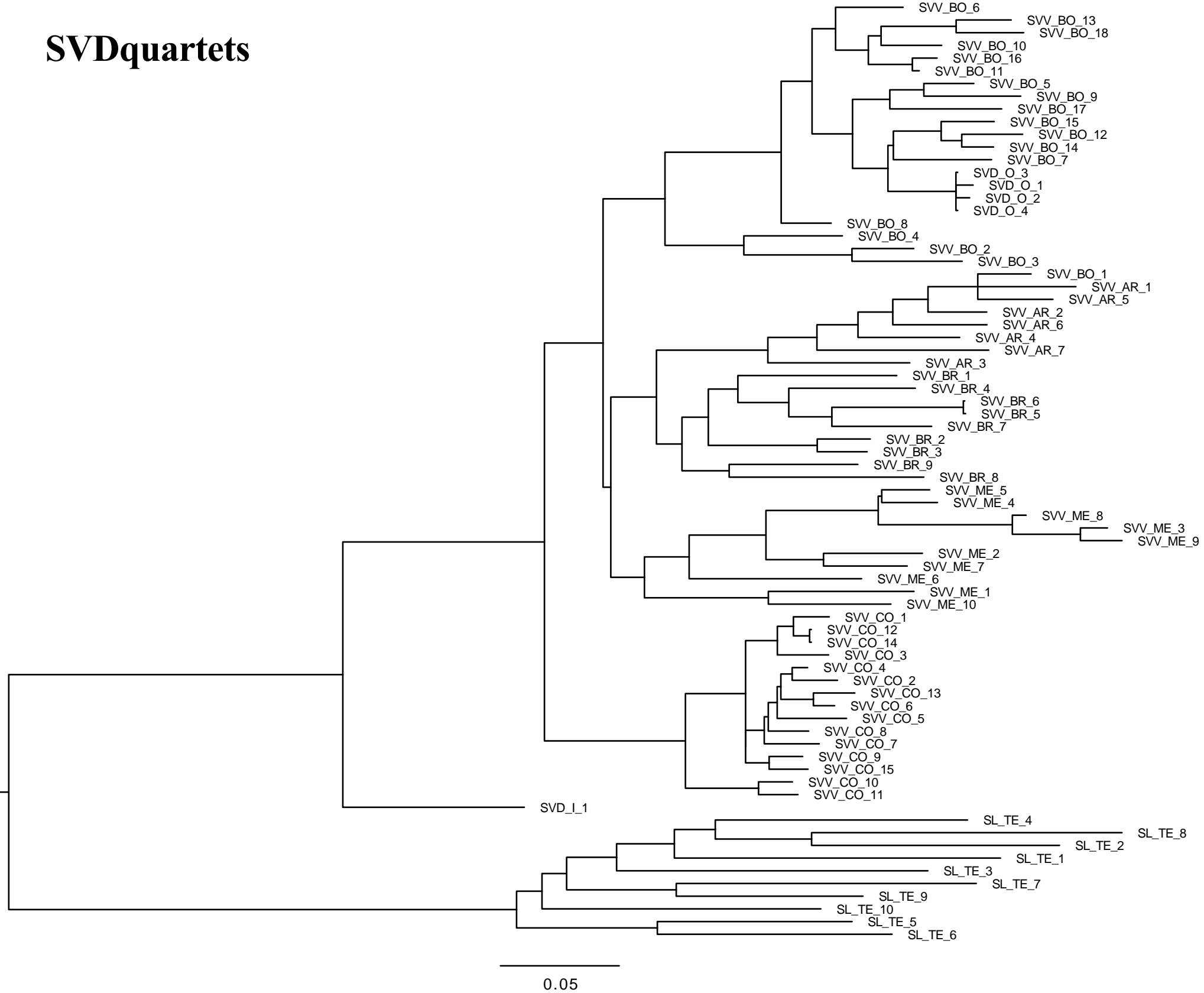

Supplement: DATA S7 — SVDquartets dendrogram. [file Data_Sheet_1.PDF]
